# O02 Beyond borders: a global risk analysis of XDR *Salmonella* Typhi and typhoid outbreaks in Pakistan

**DOI:** 10.1093/jacamr/dlae136.002

**Published:** 2024-08-23

**Authors:** Saeed Ahmad, Fahmeeda Idrees

**Affiliations:** IPPE, Tampere University, Tampere, Finland; Health Services Academy, Islamabad, Pakistan

## Abstract

**Background:**

Pakistan has experienced a surge in typhoid outbreaks caused by an emergent strain of XDR *Salmonella* Typhi since 2016. This strain, resistant to multiple antibiotics including third generation cephalosporins, poses a significant public health threat not only within Pakistan but also globally due to international travel. Understanding the mechanisms of transmission and identifying high-risk regions for importation and onward transmission of XDR *S.* Typhi are crucial for effective outbreak management and containment efforts.

**Objectives:**

This study investigates the global risk posed by XDR *S.* Typhi outbreaks, with a focus on understanding the dynamics of transmission and identifying regions at heightened vulnerability. The research aims to assess the association between air travel patterns from Pakistan and the spread of XDR *S.* Typhi globally, providing insights for targeted public health interventions.

**Methods:**

Using data from the District Health Information System (DHIS-2) for reporting priority infectious diseases in Pakistan, this study employs a combination of epidemiological analysis and phylogeographic modelling. Air travel volume data from Pakistan are analysed to discern patterns of XDR *S.* Typhi transmission to other countries. Phylogeographic models are fitted to identify associations between air travel volume and the movement of the H58 haplotype of *S.* Typhi. Flight itinerary data are cross-referenced with model-based estimates of typhoid fever incidence to identify countries at highest risk of importation and sustained transmission of XDR *S.* Typhi.

**Results:**

The analysis reveals a significant association between air travel volume from Pakistan and the rate of between-country movement of XDR *S.* Typhi, particularly the H58 haplotype. Countries with strong travel links to Pakistan and efficient local *S.* Typhi transmission are identified as being at highest risk of importation and sustained onward transmission of XDR *S.* Typhi. These findings underscore the importance of targeted public health interventions in high-risk countries to mitigate the spread of XDR *S.* Typhi.

**Conclusions:**

This study highlights the global threat posed by XDR *S.* Typhi outbreaks originating in Pakistan. The identification of high-risk countries based on air travel patterns and transmission dynamics provides valuable insights for prioritizing public health activities to track and mitigate the spread of XDR *S.* Typhi. Efforts to strengthen surveillance, enhance antimicrobial stewardship and improve sanitation infrastructure are critical for preventing and controlling future outbreaks of XDR typhoid on a global scale.

